# El Niño Southern Oscillation as an early warning tool for malaria outbreaks in India

**DOI:** 10.1186/s12936-017-1779-y

**Published:** 2017-03-20

**Authors:** Ramesh C. Dhiman, Soma Sarkar

**Affiliations:** National Institute of Malaria Research (ICMR), Dwarka Sector 8, Delhi, 110077 India

**Keywords:** ENSO, ISMR, Malaria, Early warning, Correlation coefficient

## Abstract

**Background:**

Risks of malaria epidemics in relation to El Niño and Southern Oscillation (ENSO) events have been mapped and studied at global level. In India, where malaria is a major public health problem, no such effort has been undertaken that inter-relates El Niño, Indian Summer Monsoon Rainfall (ISMR) and malaria. The present study has been undertaken to find out the relationship between ENSO events, ISMR and intra-annual variability in malaria cases in India, which in turn could help mitigate the malaria outbreaks.

**Methods:**

Correlation coefficients among ‘rainfall index’ (ISMR), ‘+ winter ONI’ (NDJF) and ‘malaria case index’ were calculated using annual state-level data for the last 22 years. The ‘malaria case index’ representing ‘relative change from mean’ was correlated to the 4 month (November–February) average positive Oceanic Niño Index (ONI). The resultant correlations between ‘+ winter ONI’ and ‘malaria case index’ were further analysed on geographical information system platform to generate spatial correlation map.

**Results:**

The correlation between ‘+ winter ONI’ and ‘rainfall index’ shows that there is great disparity in effect of ENSO over ISMR distribution across the country. Correlation between ‘rainfall index’ and ‘malaria case index’ shows that malaria transmission in all geographical regions of India are not equally affected by the ISMR deficit or excess. Correlation between ‘+ winter ONI’ and ‘malaria case index’ was found ranging from −0.5 to + 0.7 (p < 0.05). A positive correlation indicates that increase in El Niño intensity (+ winter ONI) will lead to rise in total malaria cases in the concurrent year in the states of Orissa, Chhattisgarh, Jharkhand, Bihar, Goa, eastern parts of Madhya Pradesh, part of Andhra Pradesh, Uttarakhand and Meghalaya. Whereas, negative correlations were found in the states of Rajasthan, Haryana, Gujarat, part of Tamil Nadu, Manipur, Mizoram and Sikkim indicating the likelihood of outbreaks in La Nina condition.

**Conclusions:**

The generated map, representing spatial correlation between ‘ + winter ONI’ and ‘malaria case index’, indicates positive correlations in eastern part, while negative correlations in western part of India. This study provides plausible guidelines to national programme for planning intervention measures in view of ENSO events. For better resolution, district level study with inclusion of IOD and ‘epochal variation of monsoon rainfall’ factors at micro-level is desired for better forecast of malaria outbreaks in the regions with ‘no correlation’.

## Background

Malaria is a major public health problem in India, distributed in all the 36 States and Union Territories. The western part is outbreak prone, while the central and eastern parts are endemic to malaria. It is a proven fact that malaria is most sensitive to climatic parameters [1–7]. Application of rainfall for early warning of malaria has been attempted in India dating back to 1921 [8], which continued to be used by the Government of Punjab till 1946 [9]. Owing to the changes in malariometric indices and sociological development, the indices used for early warning are obsolete now-a-days. In the year 1994, the usefulness of ‘El Niño and the Southern Oscillation’ (ENSO) in early warning of malaria outbreaks was found for the first time in India [10]. ENSO is an atmospheric-cum-oceanic phenomenon that develops over Pacific Ocean. It’s warm (El Niño) and cold (La Niña) phases result into significant climatic anomalies worldwide [11]. Since 1994, many countries have found the relationship between ENSO and malaria outbreaks [12–19] but in India, there is no revisit on the subject. ENSO has been found to have strong link with Indian Summer Monsoon Rainfall (ISMR) [20], based on which early forecast of malaria can also be possible. India being a country with vast geographical area, it is known that all regions don’t have the same effect of ENSO on malaria. Therefore, the present study was undertaken to assess the link between ENSO, ISMR and malaria outbreaks at state level in India. The generated knowledge would be useful in early warning of malaria outbreaks for early preparedness and response by the National Vector Borne Disease Control Programme (NVBDCP) for containment of outbreaks.

## Methods

### Data

Data on annual state-wise malaria incidence of India was extracted from ‘National Health Profile reports’ downloaded from the Central Bureau of Health Intelligence (CBHI [21] for years 1994–2010, and for years 2011–2015, malaria data was downloaded from National Vector Borne Disease Control Programme (NVBDCP) [22]. Monthly Oceanic Nino Index (ONI) values at Niño 3.4 regions (170°E to 120°W longitude and 5°N to 5°S) for the corresponding period were downloaded from Climate Prediction Center, Center for Weather and Climate Prediction, NOAA, USA(CPC-NOAA) [23]. State-wise mean ISMR data (1951–2000) and monthly rainfall data (1994–2015) were extracted from ‘Annual Summary reports’ and ‘Monsoon Reports’ downloaded from Indian Meteorological Department (IMD, Pune) [24].

### Data processing

As the malaria cases across the different states of India vary greatly, ‘malaria case index’ that can bring them in single measurable scale compatible to ONI index was calculated to ease the analysis. For the purpose, the ‘malaria case index’ defined by ‘relative change of no. of cases from mean’ was calculated by:

1 $${\text{Malaria}}\;{\text{case}}\;{\text{index = }}\frac{{{\text{x}}\ - {\dot{\text{x}}}}}{{{\dot{\text{x}}}}}$$

where *x* implies annual malaria cases of a year, and ${\dot{\text{x}}}$ implies average annual malaria cases of the state (1994–2015).

Similarly ‘rainfall index’ defined by ‘percentage rainfall deviation from mean’ was calculated, whereby rainfall during ISMR period i.e., June, July, August and September was considered in the study as they cover 80% of rainfall received by the country,

2 $${\text{Rainfall}}\;{\text{index}}= \frac{{{\text{x}}-{\upmu }}}{{\upmu }} \times 100$$

where *x* implies ISMR (JJAS) of a year, and *µ* implies mean ISMR (1951–2000).

Studies undertaken in India also have found that ONI value during the pre-monsoon season do not have any predictive value for ensuing monsoon rainfalls [20]. In northern hemisphere, El Niño event peaks during winters [23], therefore, the winter ONI values were considered for correlation with ISMR in the following months (concurrent ISMR) in India. For the purpose of correlation with various attributes, four month average positive ONI values (+ winter ONI) from November to February were extracted.

### Data analysis

In order to determine the impact of El Niño on malaria, a three-tier analysis was conducted, whereby, in step one, correlation coefficients (r) between ‘rainfall index’ and ‘+ winter ONI’ was determined, followed by, the correlation between ‘malaria case index’ and ‘rainfall index’, and ‘malaria case index’ and ‘+ winter ONI’. The resultant correlation between ‘malaria case index’ and ‘+ winter ONI’ was used on geographical information system (GIS) platform to generate spatial correlation map. ‘Natural Neighbour’ algorithm was used for spatial interpolation of the entire data set. This method allows the creation of highly accurate and smooth surface models from very sparsely distributed datasets [25]. The ‘r’ value greater than 0.2 and less than −0.2 were considered as significant correlation at p < 0.05 [26, 27].

## Results

### Correlation between ‘+ winter ONI’ and ‘rainfall index’

**Fig. 1:**
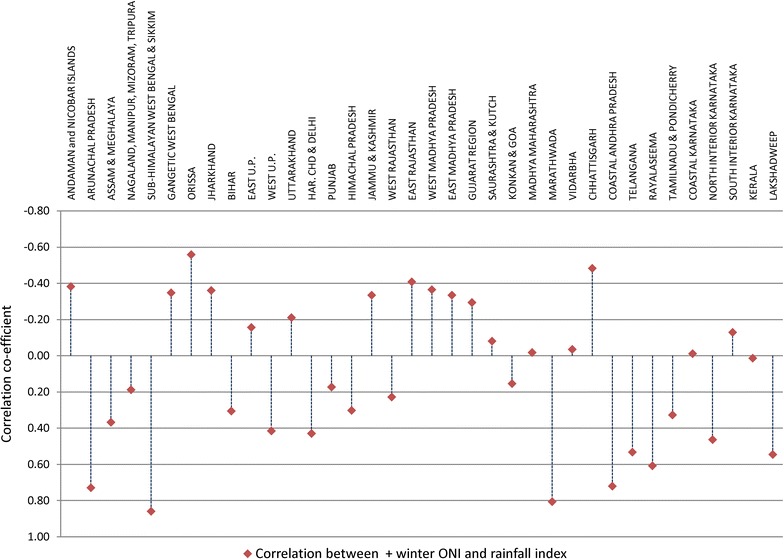
Relationship between ‘+ winter ONI’ and ‘rainfall index’ across India

To gain the insight on the relationship between El Niño and ISMR, Fig. 1 shows the correlation between ‘+ winter ONI’ and ‘rainfall index’. The correlation shows great variability in ISMR distribution across the country. When there was strong El Niño effect during winter (NDJF), the states like Orissa, Jharkhand, Chhattisgarh, Rajasthan, Gujarat, Madhya Pradesh, Uttarakhand, Jammu & Kashmir, and Gangetic West Bengal experienced negative departure of rainfall (deficit) from its normal (±10% ISMR mean) in the concurrent ISMR period. Therefore, in these states, a negative correlation was observed between ‘+ winter ONI’ and concurrent ISMR; whereas, the states like Andhra Pradesh, Tamil Nadu, Himachal Pradesh, Haryana, Delhi and N-Eastern states experience rainfall normal or excess (positive departure of rainfall from ISMR mean), exhibiting a positive correlation between ‘+ winter ONI’ and concurrent ISMR. Variability of ISMR is also found within a state e.g. variable correlation coefficient within states of East and West Uttar Pradesh, North and South Karnataka, East and West Rajasthan, Marathwada region and rest of Maharashtra.

### Correlation between ‘rainfall index’ and ‘malaria case index’

**Fig. 2:**
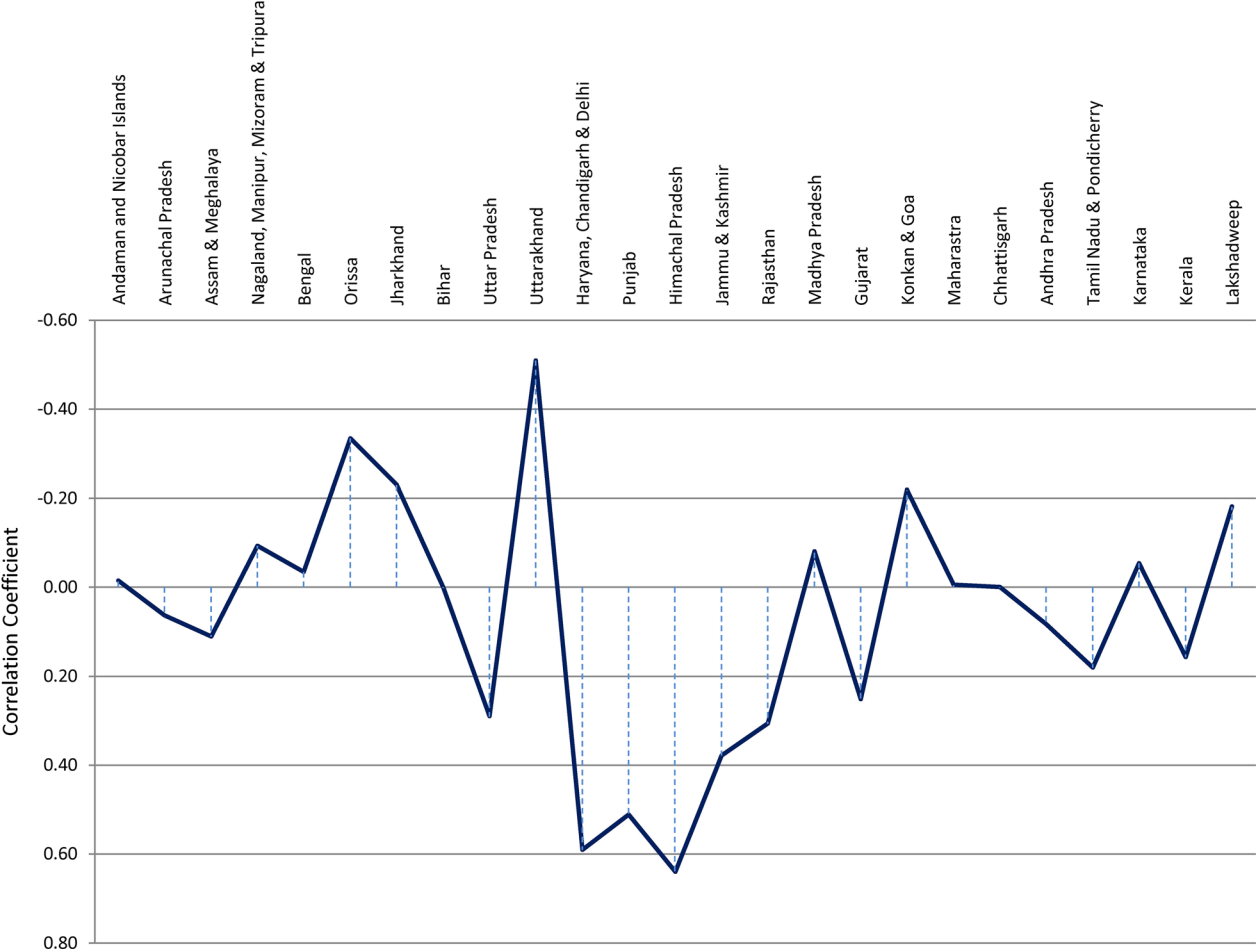
Correlation between rainfall index (JJAS) and malaria index (1994–2015)

Figure 2 shows the correlation between ISMR and ‘malaria case index’ from 1994 to 2015, showing that the states of Orissa, Jharkhand, Uttarakhand and Goa register negative correlation ranging from −0.2 to −0.5, indicating increase in malaria during ISMR deficit. The states like Rajasthan, Himachal Pradesh, Jammu and Kashmir, Haryana, Delhi, Uttar Pradesh and Gujarat register positive correlation between ISMR and ‘malaria case index’, indicating increase in malaria cases during excess ISMR. No correlation was found in the states like Bihar, Maharashtra, Madhya Pradesh, Chhattisgarh, Karnataka, Tamil Nadu, Kerala and NE states.

### Correlation between ‘+ winter ONI’ and ‘malaria case index’

**Fig. 3:**
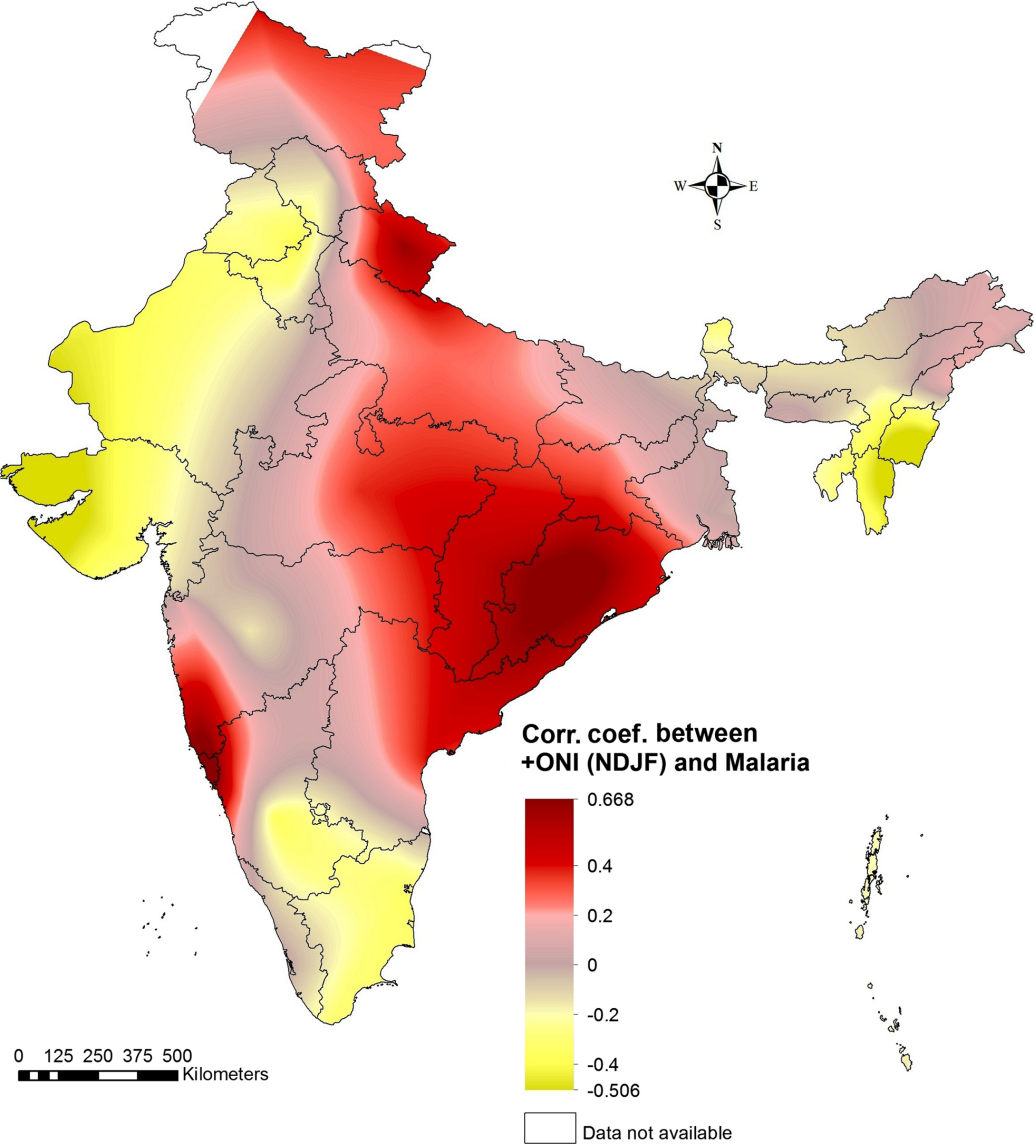
Relationship between El Niño and malaria in India

The map generated from the correlation coefficient between ‘+ winter ONI’ and ‘malaria case index’ (Fig. 3). A correlation coefficient ranging from −0.5 to 0.7 (p < 0.5) was found between ‘+ winter ONI’ and ‘malaria case index’ across the different states in India. A positive correlation indicates that increase in El Niño intensity (+ONI value) during November to February will lead to malaria outbreak in concurrent year. The states of Orissa, Chhattisgarh, Jharkhand, Bihar, Goa, eastern parts of Madhya Pradesh, part of Andhra Pradesh, Uttarakhand and Meghalaya register malaria outbreaks when there is strong ‘+ winter ONI’. Whereas, negative correlations were found in Rajasthan, Haryana, Gujarat, part of Tamil Nadu, Manipur, Mizoram and Sikkim indicating decrease in malaria cases during El Niño (+ONI), thereby during La Nina (−ONI) condition, these states are likely to face malaria outbreaks. The states like Delhi, Bihar, West Bengal, Andaman and Nicobar islands, Kerala, parts of Karnataka, Madhya Pradesh and few NE states show ‘no correlation’, i.e., no influence of ENSO events on malaria.

## Discussion

The prepared map of El Niño and malaria relationship should be viewed in the context of probabilistic ENSO outlook provided by Climate Prediction Center, NOAA at monthly interval. Present study shows that ‘winter ONI’ values are helpful in identifying the malaria outbreak-prone regions during the concurrent year in India. It supports the findings of several studies stating the evidence of the association between ENSO events and malaria conducted in Southern Africa [2, 28, 29], South Asia [30] and South America [31]. Studies have emphasized that ENSO and seasonal climate forecast might offer preparedness for epidemic control [32].

Earlier studies have also found close association between deficit ISMR and El Niño [33–35], however, understanding the malaria complexity, for predicting the malaria epidemic with respect to ENSO, regional variability of ISMR across the country has been taken into consideration in the present investigation. It shows that during El Niño phase there is variability of ISMR distribution across the country, which directly affects the malaria situation. In view of diverse geography of India, malaria situation in all geographical regions of India are not equally affected by the ISMR deficit or excess as well. In some regions rainfall deficit leads to malaria outbreaks while in other excess rainfall causes outbreaks. In drier parts, excess rainfall can create water pools and puddles; whereas in wet areas like riverine areas, deficit rainfall can result in river bed-pools, hence both are conducive for mosquito breeding, leading to malaria outbreaks in excess as well deficit rainfall scenario. Such regional variation was also put forward by others [29], where the authors concluded that regional differentiation in the role of ENSO and malaria need to be further studied for using ‘ENSO related’ climate variability into dynamic malaria models. Likewise, it has also been found that ENSO and malaria signals are not uniform throughout South America; a drought favored malaria epidemic in Columbia and Guyana with a lag period of one year, whereas, non-correlated relationship between ENSO and malaria was detected in Brazil, French Guyana, and Ecuador [31].

**Fig. 4:**
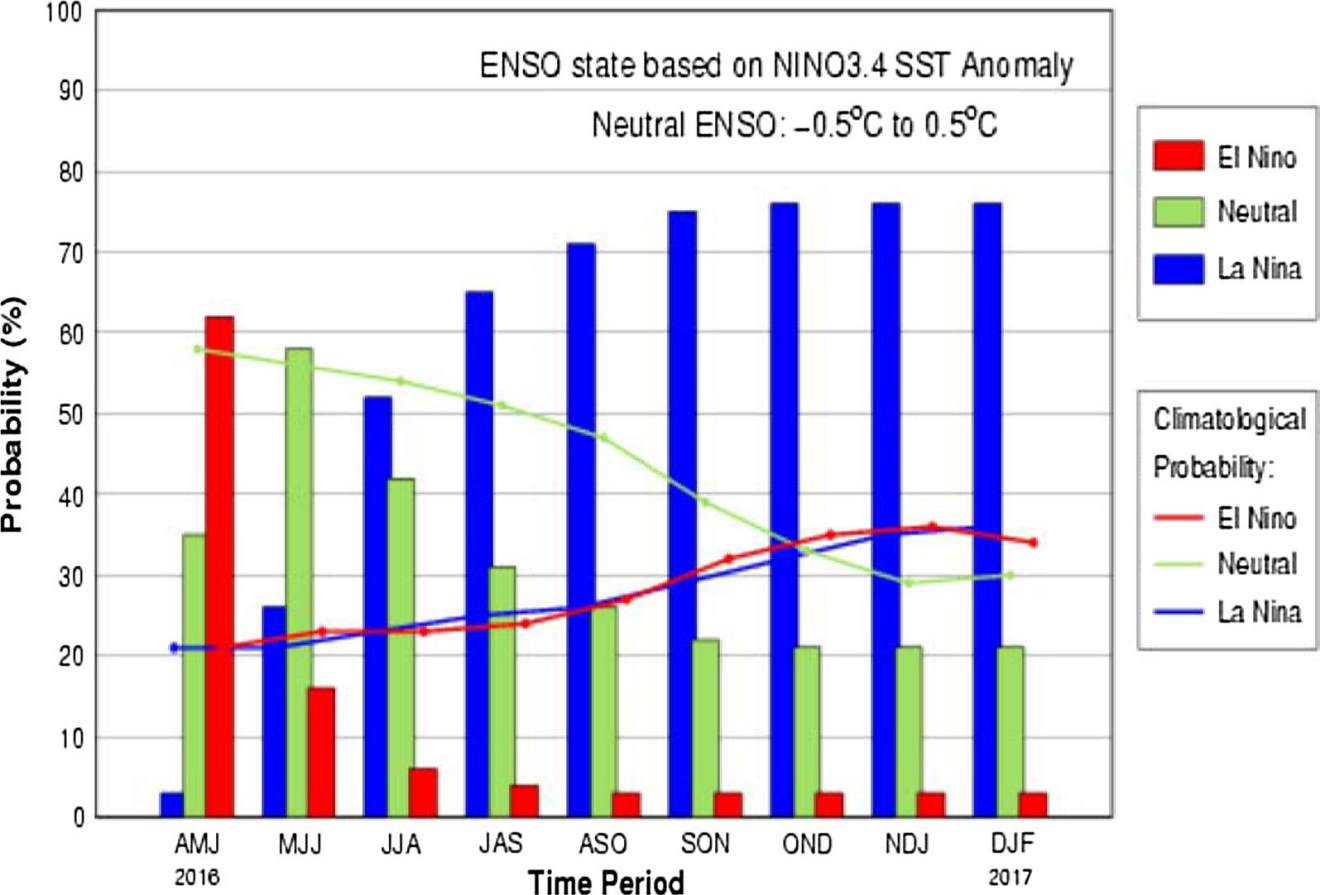
Early-May CPC/IRI Official Probabilistic ENSO Forecast (Source: http://www.cpc.ncep.noaa.gov/)

As a whole, the generated correlations between ‘+ winter ONI and rainfall index’, and ‘rainfall index and malaria case index’ corroborate the correlation between the ENSO and malaria. In India, El Niño generally suppresses monsoon rainfall. From the analysis it can be suggested that among all the states which experience ISMR deficit when there is strong‘+ winter ONI’, the states of Orissa, Chhattisgarh, Jharkhand, Bihar, Uttarakhand, and parts of Madhya Pradesh and Uttar Pradesh may register rise in malaria cases; while the states like Rajasthan, Haryana, Gujarat, part of Tamil Nadu, Manipur, Mizoram and Sikkim, which shows negative correlation are likely to experience malaria outbreaks in the event of La Nina condition. As per CPC/IRI Probabilistic ENSO Outlook of May 2016, from June onwards the chance of La Niña is roughly 75% during fall and winter 2016–17 in the Northern Hemisphere (Fig. 4). So there is high probability of above normal rainfall in later half of 2016. The states of Rajasthan, Haryana, Gujarat, part of Tamil Nadu, Manipur, and Mizoram are likely to have malaria outbreaks during 2016 provided surveillance and interventions are unchanged. As we have not considered the NE monsoon rainfall (October to December), which is about 20% of total rainfall in India and affects Andhra, Tamil Nadu coast, and interiors of Karnataka, their malaria situation cannot be forecasted from the generated map.

For the states where weak or no correlation between ENSO and malaria exists, a district level study should be conducted. Recently, the Indian Ocean Dipole (IOD) phenomenon has been found as an important manifestation that influences the relationship between Indian summer monsoon rainfall and ENSO [36]. IOD is an ocean–atmosphere coupled phenomenon in the Indian Ocean. The referred study has further found that the Indian summer monsoon rainfall (ISMR) is enhanced (decreased) during positive (negative) IOD events. Moreover, there are factors like epochal variation of monsoon rainfall, and a study has shown that the impact of El Niño on the AISMR (all India summer monsoon rainfall) was more severe during the below normal epochs than during the above normal epochs [37]. Therefore, for the areas showing no correlation in the present study, inclusion of IOD and ‘epochal variation of monsoon rainfall’ factors at district-level study can further provide prediction of malaria outbreaks in India.

The malaria outbreak forecast based on ENSO has limitations with respect to the coarse resolution, and the types of intervention measures that can change the whole scenario.

## Conclusions

The present study has put forward that moderate to strong correlation do exist between El Niño and malaria particularly in eastern part of India representing the states of Orissa, Chhattisgarh, Jharkhand, Bihar, Uttarakhand, and parts of Madhya Pradesh. While the western part representing the states of Rajasthan, Haryana, and Gujarat shows negative correlation between El Niño and malaria. Although a moderate to weak negative correlation between El Niño and malaria do exist in Tamil Nadu and interior Karnataka, in these areas rainfall also ensues during winters, therefore, ONI values for the summer months (March, April, May) should also be monitored for better understanding and forecasting of the malaria scenario in winters. The resultant map should plausibly guide the national programme in early planning of intervention measures in view of projected rainfall from July 2016 onwards. A more comprehensive study at district level is required for better understanding of the association, and generating a forewarning tool to mitigate and control of malaria outbreaks in India.

## Abbreviations

ENSO: El Niño and Southern Oscillation
ISMR: Indian Summer Monsoon Rainfall
ONI: Oceanic Niño Index
NVBDCP: National Vector Borne Disease Control Programme
CBHI: Central Bureau of Health Intelligence
CPC-NOAA: Climate Prediction Center, Center for Weather and Climate Prediction, NOAA, USA
IMD: Indian Meteorological Department, Pune
IOD: Indian Ocean Dipole
AISMR: All India Summer Monsoon Rainfall
